# Cu^2+^ Intercalation and Structural Water Enhance Electrochemical Performance of Cathode in Zinc-Ion Batteries

**DOI:** 10.3390/molecules30153092

**Published:** 2025-07-24

**Authors:** He Lin, Mengdong Wei, Yu Zhang

**Affiliations:** State Key Laboratory of Chemistry and Utilization of Carbon-Based Energy Resources, College of Chemistry, Xinjiang University, Urumqi 830017, China

**Keywords:** zinc-ion batteries, cathode materials, copper, structural water

## Abstract

This study investigates the performance of Cu-intercalated V_3_O_7_·H_2_O (CuVOH) as a cathode material for aqueous zinc-ion batteries (AZIBs). Density Functional Theory (DFT) calculations were conducted to explore the effects of Cu^2+^ incorporation and structural water on the electrochemical performance of VOH. The results indicated that Cu^2+^ and structural water enhance Zn^2+^ diffusion by reducing electrostatic resistance and facilitating faster transport. Based on these insights, CuVOH nanobelts were synthesized via a one-step hydrothermal method. The experimental results confirmed the DFT predictions, demonstrating that CuVOH exhibited an initial discharge capacity of 336.1 mAh g^−1^ at 0.2 A g^−1^ and maintained a high cycling stability with 98.7% retention after 1000 cycles at 10 A g^−1^. The incorporation of Cu^2+^ pillars and interlayer water improved the structural stability and Zn^2+^ diffusion, offering enhanced rate performance and long-term cycling stability. The study highlights the effective integration of computational and experimental methods to optimize cathode materials for high-performance AZIBs, providing a promising strategy for the development of stable and efficient energy storage systems.

## 1. Introduction

With the continuous depletion of non-renewable fossil fuels, the global energy shortage has become an increasingly urgent issue [1]. To address the energy scarcity and mitigate the environmental crises caused by conventional fossil fuels, there is an urgent need to develop and utilize renewable clean energy sources, such as wind, solar, and tidal energy, to meet the immense energy demand of human society [2,3]. However, these renewable energy sources are prone to environmental influences and suffer from intermittency and uncontrollability, which limit their stable utilization and significantly hinder large-scale development and practical applications [4]. Therefore, the development of efficient energy storage and conversion media, along with the establishment of suitable electrochemical energy storage systems, is essential to facilitate the effective utilization of renewable energy [5].

Among various energy storage technologies, lithium-ion batteries (LIBs) have been widely applied in mobile electronic devices, such as smartphones, and in the new energy electric vehicle industry due to their high energy density and high operating voltage [6,7,8]. These advances represent a significant breakthrough in current energy storage technology. However, LIBs encounter several challenges. Firstly, the relatively low abundance of lithium in the Earth’s crust results in high extraction costs. Secondly, the environmental impact and flammability of organic electrolytes pose significant safety risks during battery operation [9]. These challenges greatly restrict the further development and application of LIBs, necessitating the exploration of alternative energy storage technologies that are more sustainable and safer.

From the perspective of long-term green and sustainable development, the need for the development of low-cost and high-safety new rechargeable secondary batteries has become evident. In recent years, rechargeable multivalent metal-ion batteries, such as Mg^2+^ [10], Zn^2+^ [11], Ca^2+^ [12], and Al^3+^ [13] batteries, have attracted considerable attention due to their significant cost advantages. These metals are abundant in the Earth’s crust, effectively mitigating the risks associated with the shortage of lithium resources [14]. Furthermore, in multivalent metal-ion batteries, multi-electron transfer reactions enhance the theoretical capacity of these metal-ion batteries [15].

Among various multivalent metal-ion batteries, aqueous zinc-ion batteries (AZIBs) have drawn particular attention due to the unique characteristics of zinc as the negative electrode material. On one hand, zinc metal offers a high theoretical specific capacity of 820 mAh g^−1^, while on the other hand, its moderate potential of −0.76 V (vs. SHE) provides a combination of low cost, high safety, and high-performance energy storage [16,17,18,19,20]. Additionally, compared to non-aqueous electrolytes, aqueous electrolytes have significantly higher ionic conductivity, reaching up to 1 S cm^−1^, which contributes to the high-rate performance of the battery [21]. In terms of industrial production, AZIBs have an inherent advantage over LIBs that use expensive and flammable organic electrolytes. The manufacturing process of AZIBs does not require strict control of oxygen and moisture environments, thus simplifying the production process and reducing costs [22]. As a result, AZIBs show great potential for large-scale applications in electric vehicles and grid energy storage systems [23,24,25,26].

The cathode materials in AZIBs, as hosts for Zn^2+^ storage, directly control the working voltage, reversible capacity, and overall energy storage characteristics of the battery. Therefore, the development of high-performance cathode materials is crucial for improving the efficient energy storage and practical application of AZIBs. Current cathode materials that have been reported include vanadium-based compounds [27,28,29,30], manganese-based oxides and their derivatives [31], organic conjugated polymers [32], and Prussian blue analogs (PBAs) [33,34,35,36].

In recent years, vanadium-based compounds have garnered widespread interest for their high specific capacity, abundant resources, and notable economic advantages, making them the focus of extensive research in AZIBs. As a typical layered crystal structure, vanadium oxides offer efficient transport channels for Zn^2+^ insertion/extraction due to their unique layered framework and multi-valent characteristics, showcasing excellent electrochemical performance [37,38]. In earlier studies, various vanadium oxides, such as V_2_O_5_ [39], V_5_O_12_·6H_2_O [40], VO_2_ [41], V_6_O_13_ [42], ZnV_2_O_4_ [43], and LiV_3_O_8_ [44], have been widely explored and applied as cathode materials in AZIBs. Among them, V_3_O_7_·H_2_O (VOH) has gained significant attention due to its unique layered structure [45]. VOH consists of a V_3_O_8_ layered framework composed of VO_6_ octahedra and VO_5_ trigonal bipyramidal units, with adjacent layers connected by hydrogen bonds, offering good structural stability that facilitates the fast migration of Zn^2+^ ions. Additionally, the mixed valence states of V^5+^ and V^4+^ in VOH (with an average valence state of +4.67) provide rich redox active sites, further enhancing its theoretical capacity [46].

However, VOH materials still face several challenges in practical applications. Firstly, the strong electrostatic interaction between Zn^2+^ and the host framework increases the diffusion energy barrier of Zn^2+^ within the lattice, leading to slow Zn^2+^ insertion/extraction kinetics. Additionally, during the charge/discharge process, the crystal structure is prone to collapse due to the repeated insertion/extraction of Zn^2+^, which leads to capacity degradation and poor cycling stability [30]. To address this issue, researchers have proposed a modification strategy involving the co-intercalation of cations and water molecules [47]. When the introduced metal has a higher electronegativity than the vanadium element in the host, the chemical bonds formed between the metal and the oxygen atoms in the framework effectively stabilize the layered structure and reduce structural stress during charge/discharge, thus minimizing volume expansion and structural collapse [48]. More importantly, the retained interlayer water molecules, in conjunction with the intercalated metal ions, work synergistically to maintain efficient Zn^2+^ transport channels, thereby achieving simultaneous optimization of structural stability and reaction kinetics.

Motivated by these challenges, we performed Density Functional Theory (DFT) calculations to investigate the effects of Cu^2+^ incorporation and the presence of structural water on the electronic structure and electrochemical performance of the VOH system. The results revealed that in the Cu-intercalated VOH (CuVOH), the electrostatic resistance to Zn^2+^ diffusion is significantly reduced. This enhancement is attributed to the synergistic effect of Cu^2+^ pillars and structural water, which together facilitate faster and more efficient Zn^2+^ transport.

Inspired by these theoretical insights, we successfully synthesized CuVOH nanobelt structures via a one-step hydrothermal method. Cu^2+^ acts as an interlayer pillar, stabilizing the framework through Cu–O bonds and expanding the interlayer spacing to 0.226 nm, providing a pathway for fast Zn^2+^ migration. Additionally, the presence of interlayer water molecules further enhances the Zn^2+^ transport dynamics. Electrochemical tests reveal that CuVOH exhibits an initial discharge specific capacity of 336.1 mAh g^−1^ at 0.2 A g^−1^ and maintains a discharge capacity of 169.5 mAh g^−1^ after 1000 cycles at a high current density of 10 A g^−1^, with a capacity retention of 98.7%, demonstrating excellent cycling stability. The Cu^2+^ intercalation strategy significantly improves both the structural stability and the Zn^2+^ diffusion kinetics, enhancing the rate performance and cycling stability of the VOH cathode material. This study provides a new design approach for developing high-performance cathode materials for aqueous zinc-ion batteries.

## 2. Results and Discussion

### 2.1. DFT Calculations

**Figure 1:**
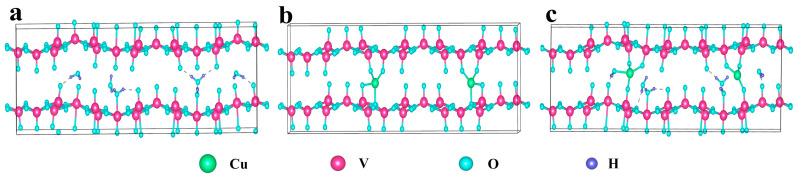
Structural configurations of (**a**) VOH, (**b**) CuVO, and (**c**) CuVOH.

We first conducted DFT calculations to explore the effects of Cu^2+^ incorporation and the presence of structural water on the electronic structure and electrochemical performance of the VOH system. The computational models consisted of the unmodified VOH layered structure (Figure 1a), CuVO with Cu^2+^ ions but without structural water (Figure 1b), and CuVOH with both Cu^2+^ ions and structural water (Figure 1c).

**Figure 2:**
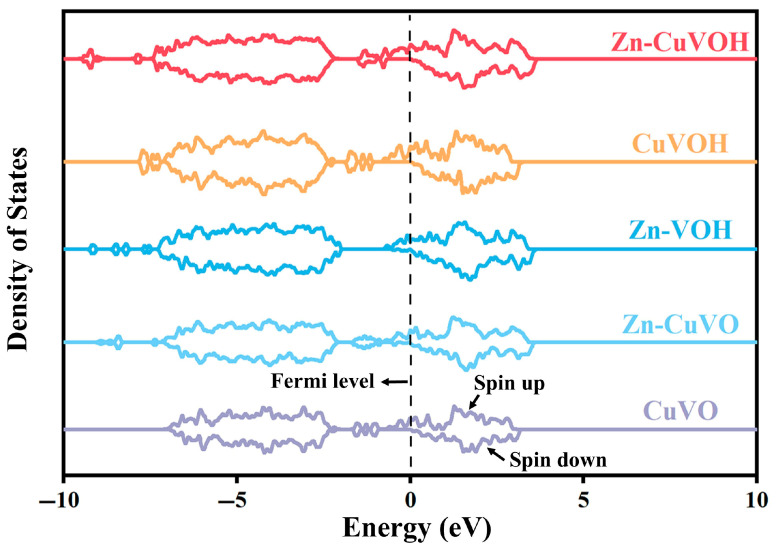
DOS of VOH, CuVO, and CuVOH.

The calculated Density of States (DOS) for these systems is presented in Figure 2, revealing significant insights into the electronic properties of each configuration. For the CuVO system without structural water, the results demonstrate a semi-metallic behavior, with a finite DOS at the Fermi level in the spin-up channel and semiconductor-like characteristics in the spin-down channel. This electronic structure suggests poor electrical conductivity, primarily due to the lack of substantial states available for conduction in the spin-down channel, which impedes efficient charge transport. The semi-metallic nature is a direct consequence of the Cu^2+^ ion introduction, which alters the local electronic structure but fails to enhance conduction sufficiently in the absence of structural water.

In contrast, the CuVOH system, which incorporates both Cu^2+^ ions and structural water, exhibits metallic behavior, as evidenced by the significantly broadened and enhanced DOS at the Fermi level for both spin-up and spin-down states. The presence of structural water serves as an electron donor, increasing the carrier concentration and optimizing the conduction pathways. This enhancement in electronic properties, attributed to the water’s role in facilitating charge transport, leads to significantly improved conductivity compared to the CuVO system without structural water.

Moreover, the introduction of Zn^2+^ into the CuVOH system results in further improvements. The Zn–CuVOH system shows a substantial broadening and increased intensity of the DOS near the Fermi level, indicating a marked enhancement in the material’s conductivity. This observation underscores the synergistic effect of Zn^2+^ incorporation, which effectively tunes the electronic structure to increase the overall carrier density. When compared to Zn-VOH, which lacks Cu^2+^ ions, the Zn–CuVOH system displays a significant increase in DOS near the Fermi level, suggesting that the CuVOH matrix provides a much more favorable electronic structure for enhanced charge transport.

**Figure 3:**
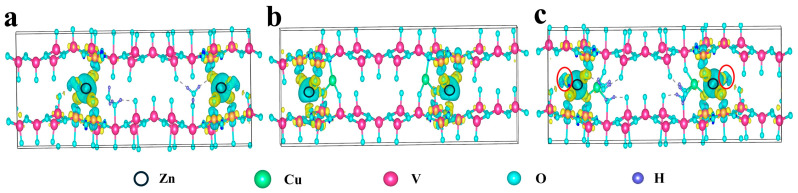
Differential charge density of (**a**) VOH, (**b**) CuVO, and (**c**) CuVOH.

In order to further elucidate the key effects of Cu^2+^ incorporation and structural water on the Zn^2+^ diffusion kinetics, we conducted a differential charge density analysis to reveal the charge transfer characteristics of different systems (Figure 3). The differential charge density maps highlight electron accumulation in yellow regions and electron depletion in blue regions, which provide crucial insights into the charge redistribution within each system. For the unmodified VOH system (Figure 3a), the differential charge density analysis indicates a significant charge rearrangement between the Zn atom and the host VOH material. The Zn atom loses a considerable amount of electrons (represented by the blue region), and these electrons predominantly transfer to the neighboring V and O atoms in the VOH structure (yellow regions). This substantial electron transfer suggests the presence of strong electrostatic interactions between Zn^2+^ and the VOH host, which are unfavorable for the efficient diffusion of Zn^2+^ ions.

In the CuVO system, which does not contain structural water (Figure 3b), the electron transfer is primarily limited to interactions between Zn and the adjacent V and O atoms, with no significant electronic changes observed near the Cu atoms. This observation suggests that the Cu^2+^ ions do not directly participate in the charge compensation process and do not significantly influence the Zn^2+^ diffusion behavior. Consequently, the lack of participation of Cu^2+^ in the charge redistribution process further hinders the diffusion of Zn^2+^ in the CuVO system.

However, in the CuVOH system, the presence of Cu^2+^ plays a pivotal role in modulating the charge distribution within the host structure. The Cu^2+^ ions facilitate charge transfer to the O atoms of the host, leading to a redistribution of electronic charge across the material. This redistribution results in the oxygen atoms acquiring additional negative charge. Consequently, when Zn atoms lose electrons, these electrons are transferred not only to the oxygen atoms of the host but also to the structural water molecules in close proximity to the host. This transfer is enabled by the altered electronic structure of the host, where the excess negative charge on the oxygen atoms reduces their ability to accept further electrons from the Zn atoms. As a result, Zn atoms preferentially transfer some of their lost charge to the structural water molecules. This redistribution weakens the strong electrostatic interactions between Zn^2+^ and the host oxygen atoms, thereby facilitating the diffusion of Zn^2+^ ions through the material. The enhanced diffusion behavior observed is attributed to the cooperative effect between Cu^2+^ and the structural water molecules, which work synergistically to promote efficient Zn^2+^ ion transport. Overall, these findings highlight that CuVOH, with its unique electronic structure and the synergistic contributions of Cu^2+^ and structural water, represents a promising candidate for high-performance zinc-ion battery applications.

### 2.2. Morphological Characterization

To verify the theoretical predictions from DFT calculations, the CuVOH material was synthesized via a one-step hydrothermal method. Quantitative elemental analysis was subsequently performed using Inductively Coupled Plasma Optical Emission Spectroscopy (ICP-OES), and the results are presented in Table 1. The ICP data indicate that the molar ratio of Cu to V in the CuVOH sample is approximately 1:17. This relatively low Cu content aligns well with the nominal stoichiometric ratio employed during synthesis, confirming the successful and controlled incorporation of Cu^2+^ ions into the VOH framework.

**Figure 4:**
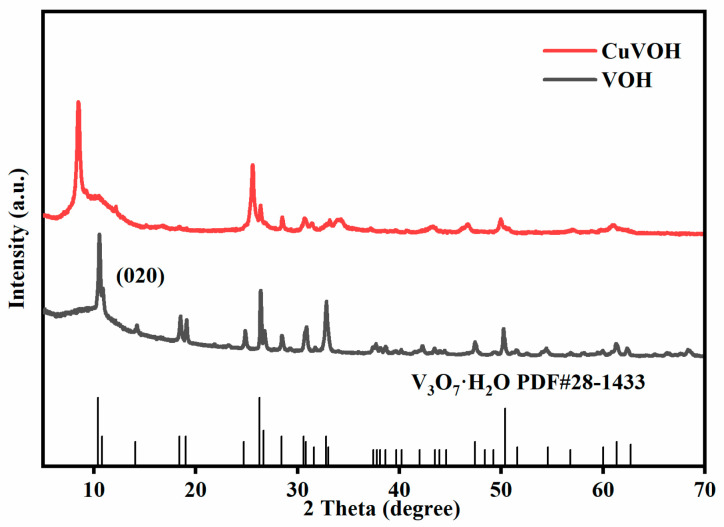
XRD patterns comparison of VOH and CuVOH samples.

The crystal structures of VOH and CuVOH were characterized by X-ray diffraction (XRD), as shown in Figure 4. The diffraction pattern of CuVOH closely matches that of the V_3_O_7_·H_2_O phase (JCPDS No. 28-1433), indicating that the introduction of Cu^2+^ does not disrupt the original layered framework. Notably, a leftward shift in the (020) diffraction peak is observed in CuVOH compared to VOH, suggesting an expansion of the interlayer spacing. This shift is attributed to the incorporation of Cu^2+^ ions into the VOH lattice, which effectively enlarges the interlayer distance while preserving the structural integrity of the host material. The increased interlayer spacing is beneficial for zinc-ion storage, as it offers more accessible pathways for Zn^2+^ insertion and extraction, thereby improving the electrochemical kinetics and overall performance of the CuVOH cathode.

**Figure 5:**
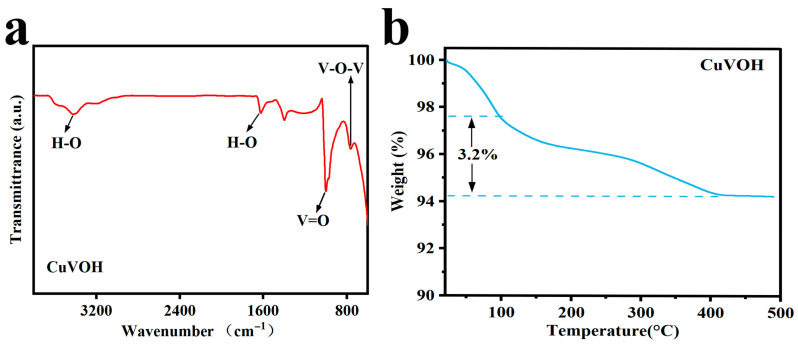
(**a**) FT-IR spectrum of CuVOH; (**b**) TGA curve of CuVOH.

**Figure 6:**
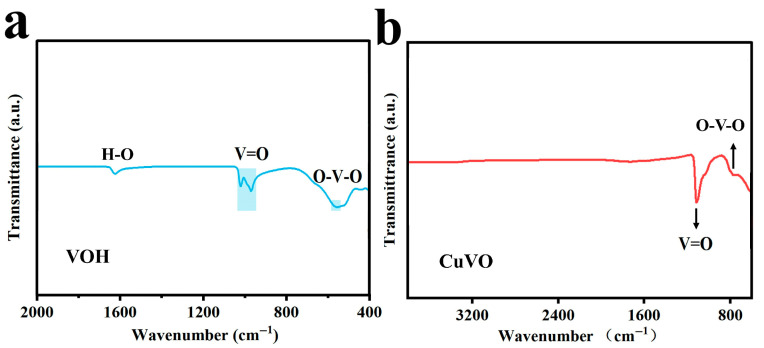
FT-IR spectrum (**a**) of VOH and (**b**) CuVO.

To gain deeper insight into the structural characteristics and hydration states of the synthesized materials, Fourier Transform Infrared Spectroscopy (FT-IR) spectra were systematically acquired for CuVOH, the parent VOH, and CuVO samples (Figure 5a and Figure 6a,b). As shown in Figure 5a, the FT-IR spectrum of CuVOH exhibits prominent absorption bands at approximately 764 cm^−1^ and 1000 cm^−1^, which can be attributed to the stretching vibrations of bridging V–O–V and terminal V=O bonds, respectively. These features are indicative of the vanadium–oxygen (VO) framework and confirm the successful retention of the VO structural motif in the CuVOH phase.

A comparative analysis with the FT-IR spectrum of the parent VOH (Figure 6a) reveals that both materials display similar vibrational features corresponding to V–O–V and V=O bonds, indicating that the incorporation of Cu^2+^ ions does not substantially disrupt the fundamental vanadium–oxygen network. Such structural consistency is essential for maintaining the electrochemical functionality of the host framework.

Beyond the framework vibrations, the FT-IR spectrum of CuVOH also displays a distinct absorption peak at 1624 cm^−1^, ascribed to the bending vibration of molecular water (H–O–H), as well as a broad band centered around 3400 cm^−1^, corresponding to the stretching vibration of O–H groups. These features provide strong evidence for the presence of interlayer or structural water molecules within the CuVOH lattice. The persistence of these water-related vibrational modes following Cu^2+^ incorporation suggests that the hydration state of the host structure remains largely preserved. This hydrated nature may play an important role in facilitating Zn^2+^ migration and enhancing the electrochemical performance of the material.

In contrast, when the FT-IR spectrum of CuVOH is compared with that of CuVO (Figure 6b), a notable difference emerges: the characteristic water-related bands at 1624 cm^−1^ and 3400 cm^−1^, observed in CuVOH, are nearly absent in the CuVO spectrum. This disappearance indicates the loss of interlayer or structural water molecules during the synthesis of CuVO. The absence of these hydration features in CuVO implies a more anhydrous structure, which could impede ionic mobility and, consequently, affect the electrochemical behavior of the material.

To quantitatively assess the water content in the CuVOH material, thermogravimetric analysis (TGA) was conducted under a nitrogen atmosphere with a heating rate of 10 °C min^−1^ over the temperature range of 30–650 °C, as depicted in Figure 5b. The TGA curve exhibits a continuous weight loss upon heating, which stabilizes above approximately 400 °C, indicating the completion of the dehydration process.

The initial weight loss below 100 °C is attributed to the removal of physically adsorbed water on the surface of the CuVOH particles. The more substantial weight loss observed between 100 °C and 400 °C corresponds to the release of coordinated or interlayer structural water, which is integrated into the crystal lattice of CuVOH. The sharp mass decrease within this temperature window confirms the presence of chemically bound water molecules, which are essential for maintaining the layered configuration and facilitating ion transport.

Based on the total weight loss in the relevant temperature intervals and the quantitative elemental analysis from ICP-OES, the chemical composition of the synthesized CuVOH material can be deduced as Cu_0.17_V_3_O_7_·0.5H_2_O.

**Figure 7:**
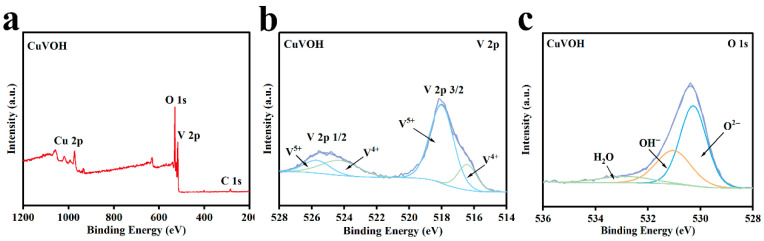
(**a**) XPS full spectrum of CuVOH; (**b**) V 2p fine spectrum of CuVOH; (**c**) O 1s fine spectrum of CuVOH.

To further verify the incorporation of Cu^2+^ and investigate the oxidation states of vanadium in the CuVOH material, X-ray photoelectron spectroscopy (XPS) analysis was performed (Figure 7a). The XPS spectra display distinct peaks corresponding to the binding energies of V, O, and Cu, confirming the successful incorporation of Cu^2+^ into the structure. The V 2p fine XPS spectrum, shown in Figure 7b, reveals peaks at binding energies of 517.4 eV and 516.1 eV, which are attributed to the presence of V^5+^ and V^4+^, respectively. The incorporation of Cu^2+^ induces a slight reduction of V^5+^ to V^4+^, leading to a mixed-valence state (V^4+^/V^5+^) that significantly enhances the material’s electronic conductivity and electrochemical activity.

Additionally, the O 1s spectrum in Figure 7c exhibits peaks at binding energies of 530.2 eV, 531.1 eV, and 532.6 eV, which are assigned to lattice oxygen (O^2−^), surface-adsorbed oxygen (OH^−^), and water molecules, respectively. These observations further confirm the presence of structural water in the CuVOH material, which aligns with the findings from FT-IR and TG analyses. The structural water provides a more favorable electrostatic environment for Zn^2+^ transport, thereby improving the electrochemical performance of the material.

**Figure 8:**
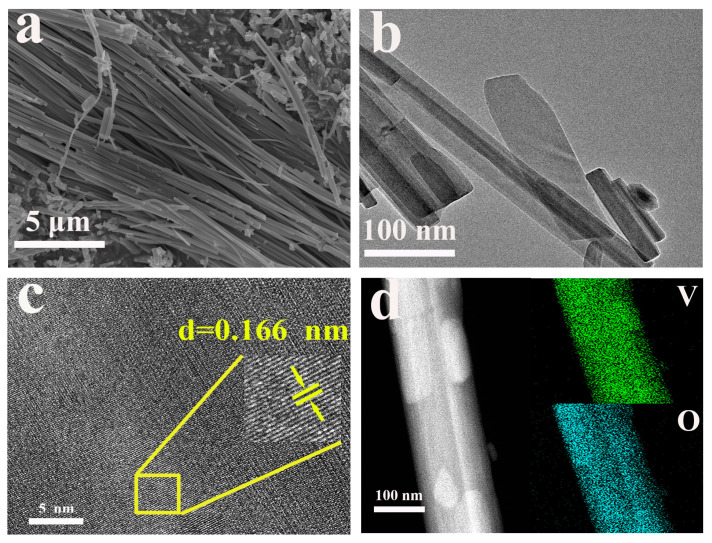
(**a**) SEM image of VOH; (**b**) TEM images of VOH; (**c**) HRTEM images of VOH and (**d**) corresponding elemental mapping.

Scanning electron microscopy (SEM) and transmission electron microscopy (TEM) were employed to investigate the morphological features of VOH and CuVOH materials. As shown in Figure 8a, the SEM image of VOH reveals a uniform and elongated nanobelt structure, with widths ranging from several hundred nanometers to a few micrometers. The TEM image (Figure 8b) further illustrates the characteristic nanobelt morphology of VOH, providing a clear visualization of the material’s structure. Additionally, the high-resolution transmission electron microscopy (HRTEM) image (Figure 8c) reveals the crystalline structure of VOH, with a lattice spacing of 0.166 nm. This measurement corresponds to the (020) plane of the material, confirming the well-defined crystalline nature of the VOH structure. The elemental distribution map (Figure 8d) indicates a homogeneous distribution of V) and O elements along the nanobelts, suggesting the uniformity of the material’s composition.

**Figure 9:**
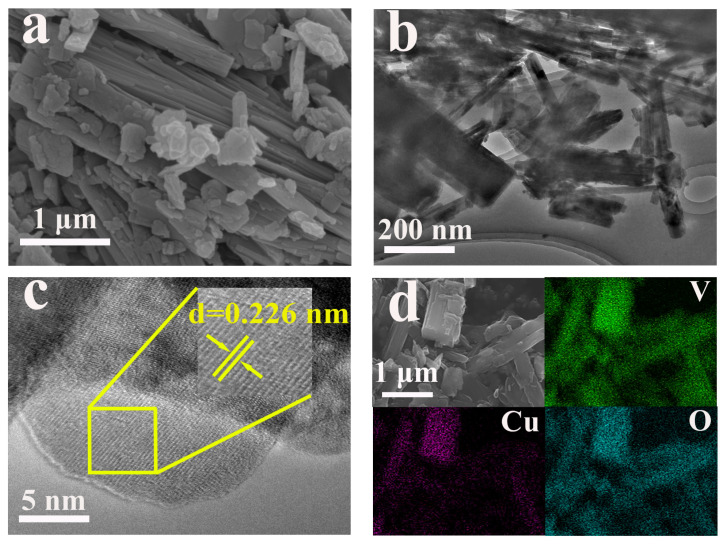
(**a**) SEM image of CuVOH; (**b**) TEM images of CuVOH; (**c**) HRTEM images of CuVOH and (**d**) corresponding elemental mapping.

Upon the incorporation of Cu^2+^, significant changes in the morphology of the material were observed. SEM images of CuVOH (Figure 9a) show a uniform yet shorter nanobelt structure compared to VOH, which can be advantageous for enhancing electron transport rates and, consequently, the conductivity of the material. The HRTEM image (Figure 9b) further provides detailed insights into the microstructure of CuVOH, with a measured lattice spacing of 0.226 nm, corresponding to the (020) plane (Figure 9c). This increase in interlayer spacing is consistent with the leftward shift of the (020) diffraction peak observed in the XRD analysis, confirming that the embedding of Cu^2+^ ions effectively expands the interlayer spacing of the material. This expansion is crucial for improving the electrochemical performance of CuVOH, as it facilitates ion diffusion and enhances charge transport.

Furthermore, the elemental distribution map (Figure 9d) of CuVOH shows a uniform distribution of Cu, V, and O elements along the nanobelts, further corroborating the successful incorporation of Cu^2+^ ions and their even distribution within the material.

### 2.3. Electrochemical Properties Characterization

**Figure 10:**
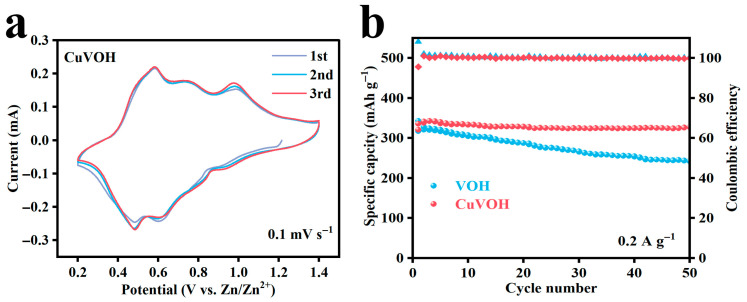
(**a**) CV curves for the first three turns of the CuVOH electrode at a scan rate of 0.1 mV s^−1^; (**b**) cycling performance plots of VOH and CuVOH electrodes at current density of 0.2 A g^−1^.

To systematically evaluate the electrochemical performance of the VOH and CuVOH materials as cathodes, CR2032-type coin cells were assembled using each material as the active electrode. The cyclic voltammetry (CV) curves of the CuVOH electrode for the initial three cycles are shown in Figure 10a. Two pairs of well-defined redox peaks are observed at approximately 0.99/0.91 V and 0.58/0.48 V, which can be attributed to the V^5+^/V^4+^ and V^4+^/V^3+^ redox couples, respectively. These results confirm that the Zn^2+^ intercalation/deintercalation process in the CuVOH structure is accompanied by multiple-step redox reactions involving vanadium ions. Moreover, the nearly overlapping CV curves across the first three cycles indicate excellent electrochemical reversibility and high structural stability of the CuVOH electrode during repeated charge/discharge processes.

The cycling performance of the VOH and CuVOH electrodes is compared in Figure 10b under a current density of 0.2 A g^−1^. The VOH electrode shows a noticeable capacity fading behavior, with an initial discharge capacity of 317 mAh g^−1^ that gradually decreases to 241.6 mAh g^−1^ after 50 cycles, corresponding to a capacity retention of only 76.2%. In contrast, the CuVOH electrode delivers a higher initial discharge capacity of 336.1 mAh g^−1^ and maintains a remarkable capacity retention of 97.4% after 50 cycles. This significant enhancement in cycling performance can be attributed to the incorporation of Cu^2+^ ions, which improves the structural integrity and charge transport properties of the electrode material. These experimental observations are in good agreement with the theoretical predictions from DFT calculations, which suggested that the introduction of Cu^2+^ and structural water facilitates better Zn^2+^ diffusion and enhances the material’s overall electrochemical stability. The superior electrochemical reversibility and long-term cycling stability of the CuVOH electrode underscore its great potential as a high-performance cathode material for AZIBs.

**Figure 11:**
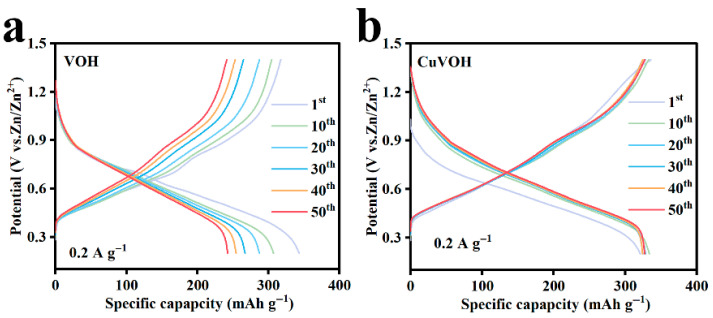
The galvanostatic charge–discharge curves of (**a**) VOH and (**b**) CuVOH for the first 50 cycles at a current density of 0.2 A g^−1^.

To further evaluate the cycling stability of the VOH and CuVOH electrodes, galvanostatic charge–discharge (GCD) measurements were conducted at a current density of 0.2 A g^−1^ over 50 cycles. As depicted in Figure 11a,b, the VOH electrode exhibits considerable variation in its charge–discharge profiles during cycling. This pronounced change in the voltage profile indicates inferior structural stability and a less reversible Zn^2+^ intercalation/deintercalation process. In contrast, the CuVOH electrode demonstrates highly overlapped GCD curves, with the 50th cycle remaining almost identical to the initial cycle. This excellent overlap provides compelling evidence of the enhanced structural stability and electrochemical reversibility of the CuVOH electrode.

**Figure 12:**
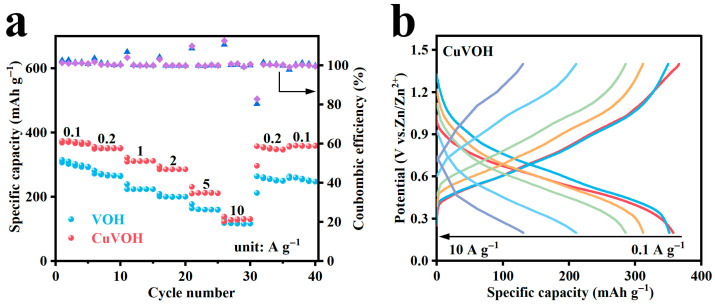
(**a**) Rate performance of VOH and CuVOH electrodes; (**b**) GCD curves of CuVOH at different current densities.

To further evaluate the rate performance of the VOH and CuVOH cathode materials, their discharge capacities were systematically tested under various current densities, as shown in Figure 12a. The CuVOH electrode exhibited outstanding rate capability, delivering high specific discharge capacities of 369, 350, 309, 286, and 128 mAh g^−1^ at current densities of 0.1, 0.2, 1, 2, 5, and 10 A g^−1^, respectively. Notably, when the current density was returned to 0.1 A g^−1^, the discharge capacity recovered to 359 mAh g^−1^, which is 97.3% of the initial capacity. This excellent capacity retention indicates superior structural stability and reversibility under high-rate conditions. In comparison, the VOH electrode showed significantly lower capacities under the same conditions, with discharge capacities of 306, 272, 224, 200, and 118 mAh g^−1^, respectively, highlighting the performance enhancement induced by Cu^2+^ incorporation.

Figure 12b presents the GCD profiles of the CuVOH electrode at various current densities. As the current density increased, the charge–discharge curves maintained a consistent shape, albeit with slightly steeper slopes, indicating limited polarization and no obvious disappearance of plateaus. This observation suggests that CuVOH maintains efficient Zn^2+^ insertion/extraction kinetics and robust structural integrity even under high-rate operation.

**Figure 13:**
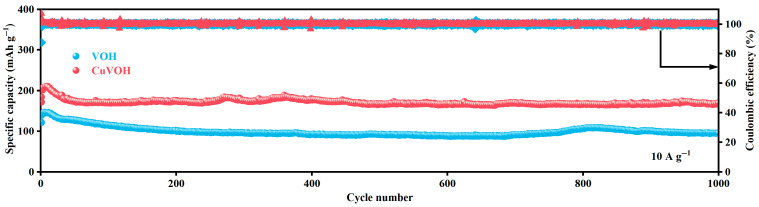
Long-cycle performance of VOH and CuVOH electrodes at a current density of 10 A g^−1^.

The long-cycle stability of electrode materials is crucial for their practical applications in batteries, particularly when high current densities are involved. To further assess the structural stability and cycling durability of CuVOH electrodes under high current densities, long-cycle performance tests were conducted. As shown in Figure 13, the CuVOH electrode exhibited exceptional electrochemical performance. The material demonstrated an initial discharge capacity of 171.8 mAh g^−1^, and after 1000 charge–discharge cycles, the specific capacity and capacity retention of CuVOH were 169.5 mAh g^−1^ and 98.7%, respectively, with a near 100% Coulombic efficiency. This highlights its remarkable long-cycle stability.

In contrast, the VOH electrode exhibited poor electrochemical stability under the same testing conditions. Its initial discharge capacity was 139.2 mAh g^−1^, but after 1000 charge–discharge cycles, the discharge capacity decreased to 94.7 mAh g^−1^, resulting in a capacity retention of only 68%. This significant deterioration further demonstrates that the unmodified VOH material suffers from poor cycling stability at high current densities.

The excellent long-cycle performance of the CuVOH can be primarily attributed to the incorporation of Cu^2+^ ions as “interlayer pillars” within the VOH structure. These Cu^2+^ ions form strong chemical bonds (Cu–O bonds) with the oxygen atoms, effectively stabilizing the layered structure and suppressing volume expansion and structural collapse during charge and discharge cycles. Furthermore, the presence of interlayer water molecules provides a smooth electrostatic environment for the rapid transport of Zn^2+^ ions, enhancing both the rate capability and cycling stability of the material.

The synergistic effect of Cu^2+^ ions and interlayer water significantly improves the long-cycle stability of CuVOH electrodes, especially under high current densities. This result further supports the findings from DFT calculations, which suggest that the structural modifications introduced by Cu^2+^ and interlayer water play a key role in enhancing the electrochemical performance of CuVOH.

**Figure 14:**
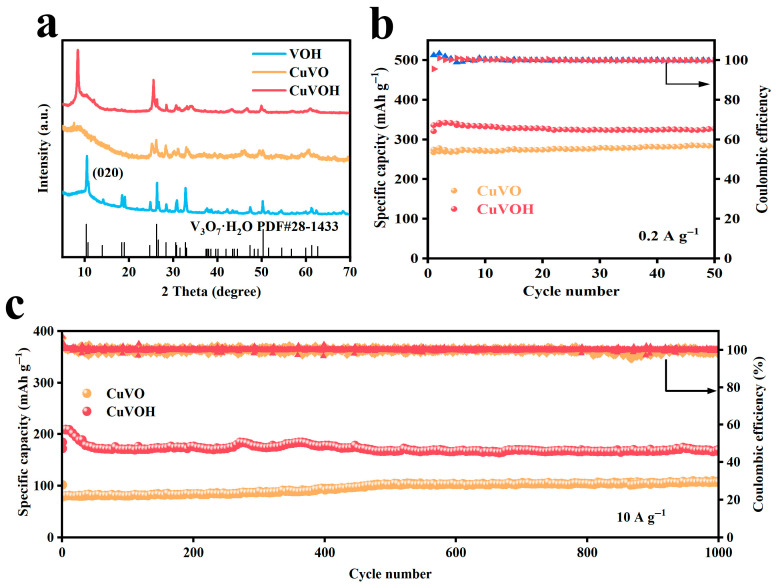
(**a**) XRD patterns of VOH, CuVO, and CuVOH; (**b**) cycling performance of CuVO and CuVOH at 0.2 A g^−1^; (**c**) long-term cycling performance of CuVO and CuVOH at 10 A g^−1^.

To further investigate the role of structural water in determining the electrochemical performance of CuVOH, a comparative study was conducted using its dehydrated counterpart, CuVO. The XRD patterns of both samples are presented in Figure 14a. Notably, the (020) diffraction peak located at approximately 8.5° exhibited a significant reduction in intensity for CuVO compared to CuVOH. This weakening of the diffraction peak is attributed to the collapse of the layered structure during the dehydration process, which compromises the structural integrity of the material. The removal of interlayer water not only alters the crystal structure but also deteriorates the stability between adjacent layers, thereby reducing the robustness of the framework.

The adverse effect of dehydration on structural stability was further reflected in the cycling performance, as shown in Figure 14b,c. At a current density of 0.2 A g^−1^, CuVOH demonstrated a high discharge capacity of 336.1 mAh g^−1^, significantly outperforming CuVO, which delivered 284.7 mAh g^−1^. More strikingly, under an ultra-high current density of 10 A g^−1^ after 1000 charge–discharge cycles, CuVOH still retained a reversible capacity of 169.5 mAh g^−1^. In contrast, CuVO exhibited a much lower capacity of 108.5 mAh g^−1^ under the same conditions, and the trend observed in the theoretical capacities for these materials is consistent with the experimental data (Table S1). These findings underscore the critical role of structural water in maintaining the layered framework and enhancing electrochemical properties. The presence of interlayer water serves as a structural stabilizer that mitigates collapse during Zn^2+^ insertion/extraction processes while also possibly facilitating ion transport and charge transfer kinetics.

**Figure 15:**
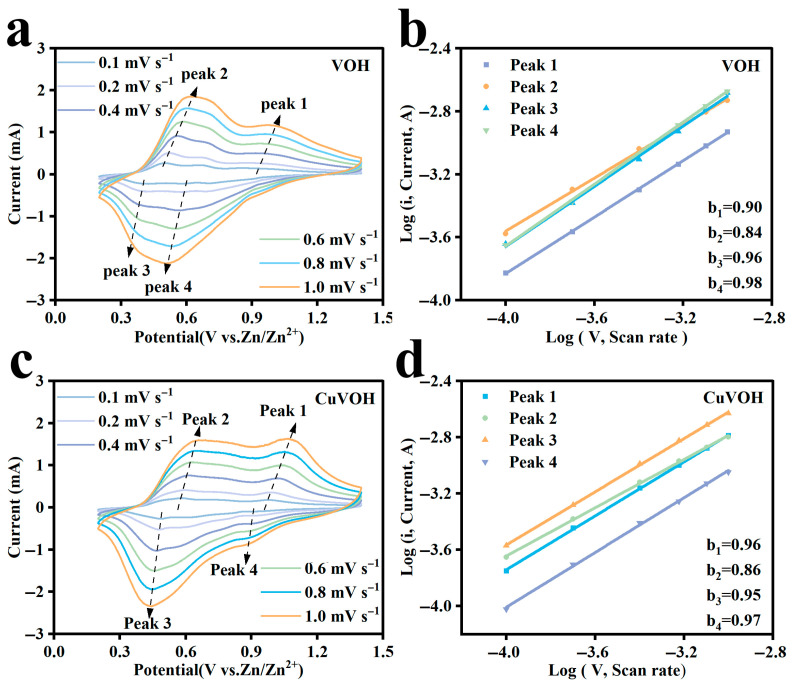
CV curves of (**a**) VOH and (**c**) CuVOH at various scan rates; log (i) vs. log (*v*) plots of the four peaks in the CV curves for (**b**) VOH and (**d**) CuVOH.

To gain a deeper understanding of the charge storage mechanism of the CuVOH cathode material, CV measurements were conducted to investigate its redox behavior. As shown in Figure 15a,c, the CV curves of both VOH and CuVOH electrodes were recorded at various scan rates ranging from 0.1 to 1.0 mV s^−1^. While both electrodes exhibit well-defined redox peaks, a shift in the peak potential (approximately 0.06 V) was observed as the scan rate increased. This indicates a partial irreversibility in the electrochemical processes, as expected in non-ideal systems.

To elucidate the kinetics of the electrochemical processes, the relationship between peak current (*i*) and scan rate (*v*) was analyzed according to the power law equation:

*i* = *av^b^* (1)

Here, the *b*-value serves as a crucial indicator of the charge storage mechanism. A *b*-value close to 1.0 suggests a surface-controlled capacitive process, while a value near 0.5 indicates a diffusion-controlled behavior. The *b*-values were obtained by fitting the linear plots of log(*i*) versus log(*v*). As shown in Figure 15b, the *b*-values for the four redox peaks of the VOH electrode fall within the range of 0.5 to 1.0, suggesting a combination of surface capacitive effects and diffusion-controlled Faradaic reactions. In contrast, the CuVOH electrode (Figure 15d) exhibits *b*-values that are much closer to 1.0, indicating a dominant capacitive behavior.

Further quantitative differentiation between capacitive and diffusion-controlled contributions was carried out using the following equation:

*i* = *k*_1_*v* + *k*_2_*v*^1/2^ (2)

Here, *k*_1_*v* represents the contribution from capacitive processes (including electrical double-layer capacitance and surface redox pseudocapacitance), while *k*_2_*v*^1/2^ corresponds to the diffusion-limited Faradaic contribution. The total current can thus be deconvoluted to determine the relative contributions of each process.

**Figure 16:**
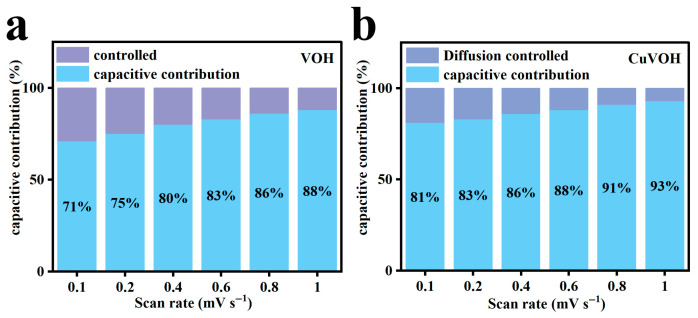
Capacitance contribution of (**a**) VOH and (**b**) CuVOH at different scan rates.

Based on this analysis, the capacitive contribution ratios at various scan rates were calculated (Figure 16a,b). For the VOH electrode, the capacitive contribution increased from 71% at 0.1 mV s^−1^ to 88% at 1.0 mV s^−1^. Notably, the CuVOH electrode demonstrated an even more pronounced capacitive dominance, with the contribution rising from 81% to 93% over the same range. This enhancement in capacitive behavior is primarily attributed to the incorporation of Cu^2+^, which improves the intrinsic electrical conductivity of the material and facilitates faster surface reactions.

The high capacitive contribution observed in CuVOH is consistent with its previously demonstrated superior rate performance, as surface-controlled processes typically enable faster electrochemical responses. These results underscore the critical role of Cu^2+^ incorporation in modulating the charge storage kinetics and enhancing the high-rate capability of vanadium-based cathode materials for zinc-ion batteries.

**Figure 17:**
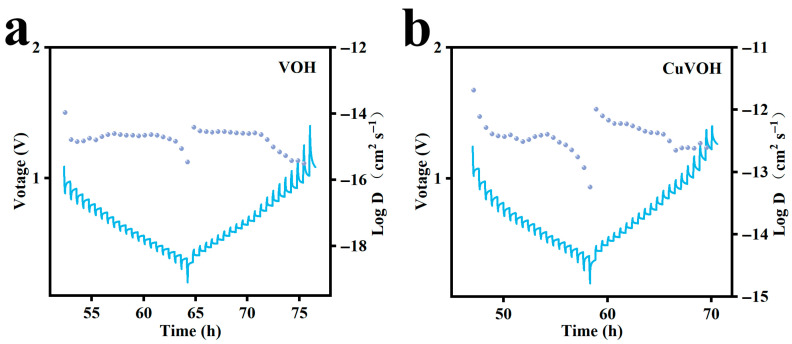
GITT curve and diffusion coefficient of Zn^2+^ at 0.2 A g^−1^; (**a**) VOH; (**b**) CuVOH.

To further elucidate the origin of the enhanced rate performance of the CuVOH cathode, the galvanostatic intermittent titration technique (GITT) was employed to quantitatively assess the Zn^2+^ diffusion kinetics, as illustrated in Figure 17. The GITT experiments were systematically conducted, consisting of a series of current pulses followed by constant current operation and concluding with a relaxation period. The experiments were carried out at a current density of 0.1 A g^−1^, with a relaxation time of 30 min. Measurements were recorded at 10 s intervals. The diffusion coefficient was calculated using the following equation:

(3) $$D^{GITT}=\frac{4}{\pi \tau }{\left(\frac{m_{B}V_{M}}{M_{B}S}\right)}^{2}{\left(\frac{{∆E}_{S}}{∆E_{t}}\right)}^{2}$$

where *τ* represents the relaxation time; *m_B_* is the mass of active material; *V_M_* denotes the molar volume; *M_B_* is the molar mass; *S* is the surface area of the electrode; and Δ*E_S_* and Δ*E_t_* are the steady-state and transient potential changes, respectively.

Using this model, the diffusion coefficients of Zn^2+^ ions were determined. For the pristine VOH electrode, the Zn^2+^ diffusion coefficients were found to range from 10^−16^ to 10^−14^ cm s^−1^. In contrast, the CuVOH electrode exhibited a significantly higher diffusion coefficient, ranging from 10^−13^ to 10^−12^ cm s^−1^, which is approximately 2–3 orders of magnitude greater than that of VOH. This substantial enhancement in ion diffusion suggests that the intercalation of Cu^2+^ ions effectively optimizes the diffusion pathways for Zn^2+^ transport within the layered structure, thereby facilitating faster ionic movement. The higher diffusion coefficients of CuVOH not only contribute to superior rate capability but also play a crucial role in suppressing undesirable side reactions during cycling. Efficient ion transport reduces the concentration polarization and ensures more uniform Zn^2+^ insertion/extraction, which in turn enhances the cycling stability of the electrode.

**Figure 18:**
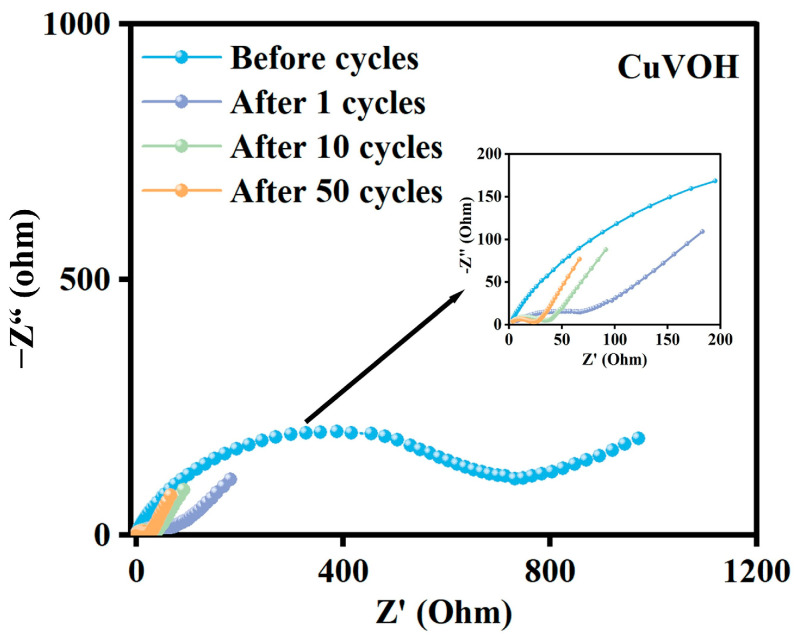
Nyquist plots of CuVOH electrode before and after cycling.

In addition, electrochemical impedance spectroscopy (EIS) was conducted before and after 50 charge/discharge cycles at a current density of 0.2 A g^−1^ to assess the interfacial charge transfer kinetics. As illustrated in Figure 18, the charge transfer resistance (*R*_ct_) of the CuVOH electrode shows a noticeable decrease after cycling, indicating an activation process during the electrochemical reactions. This reduction in *R*_ct_ reflects the improved charge transport and interfacial reaction kinetics, further supporting the enhanced electrochemical reversibility and long-term cycling stability of the CuVOH electrode.

## 3. Materials and Methods

### 3.1. Calculation Method

DFT calculations were performed using the Vienna Ab initio Simulation Package (VASP) [49]. The exchange–correlation interactions were treated using the Perdew–Burke–Ernzerhof (PBE) functional within the generalized gradient approximation (GGA) [50]. The interactions between the core and valence electrons were described by the Projector Augmented Wave (PAW) method [51,52]. A plane-wave basis set with a kinetic energy cutoff of 400 eV was employed to ensure accurate representation of the electronic wavefunctions. The convergence criteria were set to a total energy tolerance of 1 × 10^−5^ eV for the self-consistent field (SCF) calculations, and a maximum residual force threshold of 0.01 eV Å^−1^ for structural relaxation. The Brillouin zone was sampled using a Γ-centered k-point mesh of 4 × 4 × 4 for both geometry optimization and total energy calculations.

### 3.2. Preparation of Material

In the synthesis of VOH, 3.6 mmol of V_2_O_5_ was initially dispersed in a mixture of 64 mL deionized water and 2 mL acetone. The solution was stirred continuously at room temperature for 4 h to ensure complete dispersion of V_2_O_5_. The resulting mixture was then transferred to a high-pressure autoclave, which was sealed and placed in an oven for hydrothermal treatment at 180 °C for 36 h. Upon completion of the reaction, the autoclave was allowed to cool naturally to room temperature. The product was then washed alternately with deionized water and anhydrous ethanol three times to remove any residual reactants and byproducts. Finally, the product was dried under vacuum at 50 °C for 12 h to yield the VOH material.

For the synthesis of CuVOH, 3.6 mmol of V_2_O_5_ was first dispersed in a mixture of 64 mL deionized water and 2 mL acetone, and the solution was magnetically stirred at room temperature for 4 h. Subsequently, 0.1872 g of Cu(NO_3_)_2_·3H_2_O was added to the solution, and the mixture was stirred for an additional hour. The resulting solution was then transferred to a high-pressure autoclave and subjected to a hydrothermal reaction at 180 °C for 36 h. Upon completion of the reaction, the autoclave was allowed to cool naturally to room temperature. The solid product at the bottom of the autoclave was collected and washed alternately with anhydrous ethanol and deionized water to remove impurities. Finally, the washed product was dried under vacuum at 50 °C for 12 h to obtain the CuVOH material.

### 3.3. Materials Characterization

Structural characterization of the synthesized cathode materials was conducted through XRD analysis using Cu Kα radiation on a Smart Lab SE system (Rigaku Corporation, Tokyo, Japan). This method provided detailed insights into the crystalline phases, structural integrity, and potential phase transitions of the materials. The XRD patterns were carefully analyzed to identify distinct peaks corresponding to various crystalline planes, which facilitated the evaluation of material purity and crystallinity.

Morphological characterization was performed using SEM and TEM. SEM images were captured with a Hitachi SU8220 system (Hitachi, Tokyo, Japan), offering high-resolution surface imaging to examine the topography and particle distribution of the materials. Additionally, TEM analysis was carried out using an FEI Talos F200X system (Thermo Fisher, Waltham, MA, USA), providing high-magnification insights into the internal morphology and nanostructural features of the cathode materials, thereby enabling visualization of fine details at the atomic scale.

The elemental composition and distribution across the materials were further investigated using energy dispersive X-ray spectroscopy (EDS) integrated with the SEM system. This technique allowed for both quantitative and qualitative determination of the elemental composition at various locations within the samples, offering a comprehensive view of the uniformity and purity of the materials. To complement this analysis, inductively coupled plasma optical emission spectroscopy (ICP-OES) was employed with an Optima 8000 system (Pekin Elmer, Waltham, MA, USA) for precise, quantitative measurements of elemental content, including trace metals, in the cathode materials.

FT-IR spectroscopy was employed to identify the functional groups within the materials. FT-IR analyses were performed using a VERTEX 70 system (Bruker, Saarbrücken, Germany), providing detailed spectra of molecular vibrations, which provided valuable information on the chemical bonds and functional groups present. This data was crucial for elucidating the chemical composition and bonding environment of the cathode materials. XPS was utilized to examine the surface chemistry and monitor the oxidation states of the elements in the materials. XPS measurements were conducted with a Thermo ESCALAB 250Xi system (Thermo Fisher, Waltham, MA, USA).

### 3.4. Electrode Preparation and Battery Assembly

The electrode preparation process begins by mixing the active material, conductive agent (acetylene black), and binder (PVDF) in a mass ratio of 6:3:1. This mixture is then thoroughly ground for 30 min using an agate mortar to ensure uniformity. Subsequently, N-Methyl-2-pyrrolidone (NMP) solvent is slowly added to the mixture while stirring, resulting in a homogeneous slurry with an optimal viscosity. The slurry is then uniformly coated onto a 0.03 mm thick titanium foil current collector using the doctor blade coating method. The coating thickness is meticulously controlled to achieve an active material loading of 1–1.4 mg cm^−2^. Following the coating, the electrode is dried in a vacuum oven at 80 °C for 12 h to completely remove the solvent. The dried electrode is then punched into circular disks with a diameter of 10 mm using a tablet press, preparing it for battery assembly.

To evaluate the electrochemical performance of the prepared electrode material, CR2032 coin-type cells were assembled. A 0.1 mm thick zinc foil was used as the anode, and a glass fiber separator (Whatman, Maidstone, UK) was employed to separate the anode and cathode to ensure effective ionic conductivity and physical isolation. The electrolyte was a 3 M Zn(CF_3_SO_3_)_2_ aqueous solution, selected for its ability to support zinc-ion mobility during the cycling process. The prepared electrode, consisting of VOH, CuVOH, or CuVO, was utilized as the cathode in the coin cell configuration. The assembly of the coin cell was carried out in an ambient atmosphere to avoid contamination, and after assembling the components, the coin cell was sealed using a coin cell crimping machine under hydraulic pressure to ensure a secure airtight seal.

However, an important consideration during cycling is the pH fluctuations within the electrolyte. Since the electrolyte does not have significant buffering properties, pH variations are expected to occur during cycling, potentially influencing the electrochemical performance of the battery. To investigate this phenomenon, we performed a detailed pH evaluation both in the bulk electrolyte and at the interfaces near the anode and cathode regions throughout the charge–discharge cycle. pH measurements were carried out at various stages of the cycling process using pH electrodes placed strategically at the anode and cathode interfaces within the coin cell. Our results reveal that pH variations are localized primarily near the electrodes during cycling. Specifically, near the anode, the pH decreases slightly during discharge, which is consistent with the dissolution of zinc. This localized drop in pH may influence the dissolution dynamics of the zinc anode and potentially accelerate its degradation over time. However, this pH decrease did not lead to significant performance degradation of the anode within the range of pH fluctuations observed.

In contrast, pH in the cathode region increases due to the oxygen reduction reaction (ORR) and other electrochemical reactions occurring at the cathode-electrolyte interface. The increase in pH in the cathode region could affect the stability of the VOH, CuVOH, and CuVO cathode materials. Despite these pH variations, the electrochemical performance of the cathodes remained stable, with only minimal degradation in charge–discharge efficiency and cycling stability observed. Notably, the pH changes were not uniform throughout the entire cell, with more significant fluctuations occurring in the near-electrode regions. This observation suggests the development of localized pH gradients during battery operation, which could influence the electrochemical behavior at the interfaces between the electrodes and the electrolyte. These pH gradients are crucial for understanding the long-term performance of the battery, as they can impact the efficiency of ion transport and the overall stability of the electrochemical reactions at the interfaces.

Given that the electrolyte lacks sufficient buffering capacity to maintain a stable pH over extended cycling, the observed pH fluctuations may pose a limitation to the battery’s long-term cycling stability. While these variations did not result in significant degradation of electrochemical performance in the current study, it is essential to address this limitation in future studies. To mitigate the impact of pH fluctuations, we suggest incorporating pH buffers or exploring alternative electrolyte formulations with enhanced buffering properties. The addition of such buffers would help stabilize the electrolyte pH and reduce the fluctuations during cycling, thereby improving the overall cycling stability and performance of the zinc-ion battery.

### 3.5. Electrochemical Measurements

The electrochemical measurements were performed using a CHI 760E electrochemical workstation in a coin cell configuration with two electrodes. The cathode sheet, coated with the active material, was used as the working electrode. CV was carried out within a voltage range of 0.2 V to 1.6 V (vs. Zn/Zn^2+^), employing varying scan rates of 0.1, 0.2, 0.4, 0.6, 0.8, and 1 mV s^−1^ to analyze the electrochemical behavior of the electrode. The CV tests provided valuable insights into the redox reactions occurring at the electrode surface, revealing the kinetics and charge transfer characteristics of the material.

The EIS was conducted to measure the impedance of the system using a small-amplitude sinusoidal AC signal. The frequency range was set from 0.01 Hz to 100 kHz, with a voltage amplitude of 5 mV. The obtained data were analyzed using equivalent circuit modeling to evaluate the internal resistance and charge transfer resistance of the electrodes. This approach provided important information on the electrochemical behavior of the materials, especially in terms of their performance under different charge/discharge conditions.

In addition, the GITT was employed to investigate the diffusion processes and the relationship between charge transfer and electrochemical reactions at the electrode surface. This technique involved cycles of pulse application, constant current, and relaxation, allowing for the determination of the chemical diffusion coefficient. The GITT analysis provided further understanding of the material’s transport properties and its ability to facilitate ion diffusion during charge and discharge cycles.

Furthermore, electrochemical cycling and rate capability tests were performed on the coin cells at room temperature. These tests involved continuous cycling within a voltage range of 0.2 V to 1.6 V, utilizing the CT2001A Battery Test System (Wuhan LAND Electric Co, Wuhan, China). The cycling tests assessed the stability and capacity retention of the electrodes over multiple charge/discharge cycles, while the rate capability tests measured the electrodes’ performance under varying current densities. Collectively, these electrochemical measurements provided a comprehensive evaluation of the performance, stability, and transport properties of the examined electrode materials.

## 4. Conclusions

In conclusion, this study presents a comprehensive investigation into the potential of CuVOH as a high-performance cathode material for AZIBs, leveraging both DFT calculations and experimental validation. The DFT results provided valuable insights into the electrochemical behavior of CuVOH, revealing that the incorporation of Cu^2+^ ions and the presence of interlayer water molecules synergistically enhance Zn^2+^ diffusion kinetics by reducing the electrostatic resistance within the framework. This theoretical understanding guided the successful synthesis of CuVOH nano-belt structures using a one-step hydrothermal method. The experimental results confirmed the predictions from DFT calculations, demonstrating that Cu^2+^ acts as an effective interlayer pillar, expanding the interlayer spacing and stabilizing the structure, while the interlayer water molecules improve the Zn^2+^ transport dynamics.

Electrochemical tests revealed that CuVOH exhibited an impressive initial discharge capacity of 336.1 mAh g^−1^ at 0.2 A g^−1^, and after 1000 cycles at 10 A g^−1^, it maintained a high capacity of 169.5 mAh g^−1^ with a capacity retention of 98.7%. These results underscore the critical role of Cu^2+^ intercalation and structural water in enhancing both the rate performance and cycling stability of the VOH cathode material. The combined DFT-experimental approach not only validates the potential of CuVOH for high-performance AZIB applications but also highlights the synergistic effect of Cu^2+^ and interlayer water as a core innovation. This study offers new avenues for the design and optimization of advanced cathode materials in energy storage systems, contributing to the development of more efficient and sustainable batteries for large-scale applications.

## Figures and Tables

**Table 1:** ICP-OES results for the elemental composition of the synthesized CuVOH sample.

Element	Mass Content (mg kg^−1^)	Molar Amount (mmol kg^−1^)
Copper (Cu)	39,965	628.91
Vanadium (V)	567,432	11,138.89

## Data Availability

The data presented in this study are available on request from the corresponding author.

## Supplementary Materials

The following supporting information can be downloaded at: https://www.mdpi.com/article/10.3390/molecules30153092/s1, Table S1: Theoretical Capacity of Cathode Materials.
