# Supplementary material for: Cu2+ Intercalation and Structural Water Enhance Electrochemical Performance of Cathode in Zinc-Ion Batteries
Source: Molecules. 2025 Jul 24;30(15):3092. doi: 10.3390/molecules30153092 (PMC12348052; doi:10.3390/molecules30153092)
Supplement: Supplementary file 1 [file molecules-30-03092-s001.zip › molecules-3771736-supplementary.pdf]

Supporting Information

Cu^{2+} Intercalation and Structural Water Enhance Electrochemical Performance of Cathode in Zinc-Ion Batteries

He Lin, Mengdong Wei and Yu Zhang*

State Key Laboratory of Chemistry and Utilization of Carbon Based Energy Resources, College of Chemistry, Xinjiang University, Urumqi, 830017, Xinjiang, China; davisallende79@gmail.com (M.W.); cnuo017@gmail.com (Y.Z.)

*Correspondence: helin@xju.edu.cn; Tel.: +86-180-4090-9207

Table S1. Theoretical Capacity of Cathode Materials.

Cathode materials	Theoretical capacity (mAh g^{-1})
VOH	151.32
CuVO	256.25
CuVOH	297.51

The theoretical capacity of the cathode materials was calculated using the following expression:

$$C = (x_{max} v F 10^3)/M$$

where C is the theoretical capacity (in mAh g^{-1}), x_{max} is the maximum Zn^{2+} concentration that can be stored in the cathode formula unit, v = 2 is the number of valence electrons of Zn; and F is Faraday's constant (26.801 Ah mol^{-1}), M is the

molecular weight of the cathode formula unit.

The trend observed in the theoretical capacities for these materials is consistent with the experimental data, confirming that the calculated values fall within the expected range for these types of cathode materials.

However, it is important to emphasize that there is a discrepancy between the theoretical and experimental capacities. The theoretical capacities reported here are lower than the experimental values, primarily due to the following factors:

1. Faradaic vs Non-Faradaic Contributions:

The theoretical capacity calculation, as defined, only accounts for the Faradaic capacity, which arises from the redox reactions of Zn^{2+} ions in the cathode material. Specifically, this calculation is based on the transfer of electrons during the electrochemical reaction of the cathode material with Zn^{2+}.

2. Non-Faradaic Contributions:

The experimental capacity includes contributions from both Faradaic and non-Faradaic processes. The non-Faradaic contribution is mainly attributed to the electrical double-layer capacitance effect, which arises from the ability of the cathode material to store charge through electrostatic interactions at the electrode-electrolyte interface. This capacitance is not considered in the theoretical capacity calculation, which leads to the observed difference between the theoretical and experimental values.

Therefore, while the theoretical capacity calculation provides insight into the maximum possible capacity based on the Faradaic reactions, the experimental capacities reflect a more comprehensive view that incorporates additional contributions from the non-Faradaic processes. This distinction is crucial for understanding the overall electrochemical performance of the cathode materials and highlights the need to consider both Faradaic and non-Faradaic contributions when evaluating the materials in practical applications.
